# Postnatal care education in health facilities in Accra, Ghana: perspectives of mothers and providers

**DOI:** 10.1186/s12884-020-03365-1

**Published:** 2020-11-04

**Authors:** Medge D. Owen, Elizabeth Colburn, Cecilia Tetteh, Emmanuel K. Srofenyoh

**Affiliations:** 1 Department of Anesthesiology, Wake Forest School of Medicine, Medical Center Boulevard, Winston-Salem, NC 27157-1009 USA; 2 Kybele, Inc, Lewisville, NC USA; 3 Greater Accra Regional Hospital, Ghana Health Service, Accra, Ghana

**Keywords:** Postnatal care, Sub-Saharan Africa, Ghana, Low-and middle-income countries

## Abstract

**Background:**

The recent use of antenatal care (ANC) has steadily improved in low- and middle-income countries (LMIC), but postnatal care (PNC) has been widely underutilized. Most maternal and newborn deaths occur during the critical postnatal period, but PNC does not receive adequate attention or support, particularly in Sub-Saharan Africa. In Ghana, the majority of mothers attend four ANC assessments, but far fewer receive the four recommended PNC visits. This study sought to understand perceptions toward PNC counselling administered prior to discharge among both mothers and healthcare providers in the Greater Accra Region of Ghana.

**Methods:**

Facility assessments were conducted among 13 health facilities to determine the number and type of deliveries, staffing, timing of discharge following delivery and the PNC schedule. Structured interviews were conducted for 172 mothers over four-months in facilities, which included one regional hospital, four district hospitals, and eight sub-district level hospitals. Additionally, healthcare providers from 12 of the 13 facilities were interviewed. Data were analyzed with Chi-square or students t-test, as appropriate, with *p* < 0.05 considered statistically significant.

**Results:**

Ninety-nine percent of mothers received PNC instructions prior to hospital discharge, the majority of which were given in a group format. Mothers in the regional hospital were significantly more likely to have been informed about maternal danger signs but were less likely to know the PNC schedule than were mothers in district and sub-district facilities. No mother recalled more than four maternal or five newborn danger signs. Thirty-eight percent of facilities did not have PNC guidelines. Most patient and providers reported positive attitudes toward the level of PNC education, however, knowledge was inconsistent regarding the number and timing of PNC visits as well as other critical information. Only 23% of patients reported having a contact number to call for concerns.

**Conclusions:**

Despite overall positive feelings toward PNC among Ghanaian mothers and providers, there are significant gaps in PNC education that must be addressed in order to recognize problems and to prevent serious complications. Improvements in pre-discharge PNC counseling should be provided in Ghana to give mothers and babies a better chance at survival in the critical postnatal period.

**Supplementary Information:**

The online version contains supplementary material available at 10.1186/s12884-020-03365-1.

## Background

Improving access to high quality antenatal and postnatal care services has been shown to reduce maternal morbidity and mortality and improve newborn outcomes [1]. The use of antenatal care (ANC) services has steadily improved in low- and middle-income countries (LMIC) over the last decade, however, improvements in postnatal care (PNC) uptake have been slow [2, 3]. Many countries report that the majority of pregnant women receive at least one, if not more ANC visits, and a growing number of women receive four ANC visits [2–6]. This is in sharp contrast to the provision of PNC, an area which remains vastly underutilized. A study from the Democratic Republic of the Congo found that 93% of pregnant women received ANC, but only 35% received PNC [7]. Similarly, in Ethiopia, 96% of pregnant women reported having had some ANC, including 50% who claimed four visits, yet only 15% reported PNC [6]. The difference in utilization of antenatal and postnatal services is alarming since many maternal and neonatal deaths occur following delivery [2, 8]. It is estimated that between 20 and 44% of maternal deaths in low-resource settings occur during the postpartum period [2]. Seventy-five percent of newborn deaths occur during the first week of life, and newborn deaths during the first 28 days, account for nearly half of all deaths under the age of five [8, 9]. PNC remains a neglected service area that has not received the same degree of focus and programming that ANC has achieved [2, 10, 11].

The World Health Organization (WHO) recommends that mothers receive four postnatal assessments beginning within 24-h of birth and continuing at day 3, between days 7–14, and at 6 weeks [12]. The first postnatal check should be conducted in the hospital prior to discharge [12]. This assessment is essential for evaluating the mother and newborn for complications and to teach essential newborn and self-care practices [13]. Consistent with patterns noted in other sub-Saharan countries, in Ghana, 89% of women attended the four recommended ANC visits but PNC was variable; 72% received a postpartum check within 24 h of delivery, and 81% were evaluated within the first 2 days, but only 4% of women received PNC during the 3 to 41 day timeframe [9, 14]. Furthermore, only 23% of newborns in Ghana received an examination within 48 h of birth and 30% a checkup within 1 week, indicating that 70% of newborns do not receive any postnatal assessment [14]. While various programs and national insurance coverage have raised societal awareness of the necessity of ANC, there has been slower uptake of PNC [5]. To assess women’s knowledge of PNC, a qualitative study was conducted in 2015 in 40 Ghanaian mothers who either gave birth at home or in a facility [15]. Many women did not understand the various checks that occurred during PNC including the purpose of equipment used during an examination such as thermometers or stethoscopes [15]. Additionally, there was poor communication noted between health workers and mothers throughout the postnatal checks [15]. The communication deficiencies between healthcare providers and mothers as well as the general lack of knowledge of PNC throughout communities may contribute to societal perception that PNC is less important than ANC.

Little is known about the content or quality of PNC that is provided to women prior to discharge following facility birth [2, 16]. It has become clear that increasing facility-based births has not decreased maternal and neonatal mortality and high quality intrapartum and postpartum care cannot be assumed [11, 16, 17]. The postpartum assessment prior to discharge is critically important, as it may represent the only PNC evaluation that a mother or newborn receives. The purpose of this study was to gain a better understanding of the perceptions of PNC counselling received prior to discharge among Ghanaian mothers and healthcare providers at various health facilities in Greater Accra. It is hoped that the findings will be used to strengthen postnatal discharge education, improve the quality of PNC for mothers and newborns, and generate a wider demand for PNC services in Ghana.

## Methods

### Study setting

The study was conducted in the Greater Accra Region of Ghana. The region has approximately 4 million inhabitants and accounts for 16% of Ghana’s population [18]. The region is predominately urban and represents the most educated and wealthiest segment of the population [9]. The government health system is organized within a three-tier framework, whereby basic obstetric care begins in primary and community health centers but may progress to district hospitals for emergency obstetric care, and finally to regional and teaching hospitals for the highest level of care for life threatening conditions [19].

Thirteen healthcare facilities in Greater Accra were included to assess the content and retention of postnatal counselling. The Greater Accra Regional Hospital (GARH) was selected as a major obstetric referral hospital, as well as 4 district and 8 sub-district level facilities within the referral cluster. Facility assessments were initially conducted to determine the number of deliveries, cesarean delivery rate, number of midwives and PNC specific nurses, frequency of staff rotations, timing of discharge following vaginal or cesarean delivery and the schedule of PNC visits. A clinically practicing midwife from GARH, familiar with PNC requirements, was recruited and trained to administer postpartum patient and staff surveys to gain an understanding of the postnatal education format, timing and content.

### Postnatal patient interviews

Interviews were conducted among postpartum women who had delivered within a selected facility within the previous 72 h. Patients were included if they had experienced a live birth (vaginal or operative), were in good health, and were awaiting imminent facility discharge. Patients were excluded if they were in poor health or they were being discharged without their newborn. Following verbal consent and prior to hospital discharge, a structured questionnaire was administered in a semi-private setting by the trained nurse in the local dialect (Supplement 1 Exit Interview for PNC Mothers). An oral format was used to reduce potential barriers of illiteracy and lack of familiarity with medical terminology. Questions included age, parity, name of delivery facility, whether the patient had been informed about the importance of postnatal care during an antenatal visit, the postnatal counseling format, what information was provided, to whom, the number and timing of follow-up postnatal visits, what maternal and newborn danger signs and other conditions to be aware of, whether a contact phone number was given and suggestions for improvement. Participation was voluntary and patient anonymity was maintained.

### Key informant interviews

During the same time intervals, key informants from each health facility were interviewed regarding their views of the postnatal educational process (Supplement 2 Interview for PNC Providers). Informants included various grades of midwives or nurses who were in positions of leadership within their facilities and familiar with the postpartum educational process. Questions addressed the availability of PNC guidelines, adherence to the guidelines, the educational format used, the schedule for PNC visits, whether mothers were encouraged to call for questions or concerns, what barriers or challenges are faced, and what strategies or interventions could improve the process.

### Data analysis

Data from facility assessments, patient and key informant interviews were manually recorded in English on a data sheet. Data was keyed into Microsoft Excel (Microsoft, Redmond, WA) and verified prior to analysis. Data were analyzed with Chi-square or students t-test (Social Science Statistics, 2020), as appropriate, with *p* < 0.05 considered statistically significant. Institutional review board approval was granted by the Ghana Health Service (GHS/DGS/K-6/18 September 2017) and Wake Forest University Health Sciences (IRB00047565).

## Results

### Facility assessments

Facility assessments were conducted March 8–21, 2017. The number of deliveries at each facility was collected for the previous 2 years. The average number of deliveries and staffing characteristics across facility types are shown in Table 1. Cesarean deliveries were only conducted in regional and district hospitals. Every regional and district level hospital had dedicated postnatal nurses, but in three of the eight sub-district level hospitals, the same labor ward midwifes doubled as postnatal nurses. In all of the district hospitals, staff rotation occurred annually, except in one facility where rotation occurred biennially. In sub-district level hospitals, rotation of staff occurred annually in five hospitals and did not occur in three hospitals nor did it occur at the regional hospital. Regarding timing of discharge, in the regional hospital, discharge occurred 12–24 h after vaginal delivery and 2–3 days after operative delivery for low risk women. In the district hospitals, the timing of patient discharge occurred 6 h after vaginal delivery in the three higher volume (> 3000 deliveries) and 24 h after vaginal delivery in the one lower volume (< 2000 deliveries) hospital. For operative delivery, two district hospitals discharged patients after 48 h and two after 72 h of delivery. For sub-district level hospitals, patients were discharged 6 h following vaginal delivery in three hospitals and at 24 h in five hospitals, without regard to delivery volume.

**Table 1:** Hospital characteristics and staffing

	2015		2016		2016	
Facility type	Deliveries	CS rate	Deliveries	CS rate	Labor ward midwives	Postnatal nurses
Regional	8566	48%	7824	49%	34	12
District	2808 (1926-3289)	18% (2–30%)	2935 (1925-3258)	22% (13–25%)	16 (12–21)	6 (2–10)
Sub-district	1307 (503–2949)	–	1330 (559–2935)	–	15 (7–26)	4 (1–9)

Data are presented as number, average or percent (range). Cesarean section (CS). Sub-district hospitals do not conduct cesarean deliveries. All but three sub-district facilities had postnatal nurses

### Postnatal interviews

Between August 30, 2017 and November 28, 2017, 172 structured postnatal patient interviews were conducted in the 13 selected health facilities in the Greater Accra region. There were 21 (12%) interviews at the regional hospital, 68 (39%) at four district hospitals and 83 (48%) at eight sub-district level hospitals. Overall, fifty-eight (34%) interviews were done for new mothers. Patient demographic and educational level data within hospital categories is shown in Table 2. Over half (57%) the patients were made aware of the need for PNC during their ANC visits and this was similar across hospital types (Table 3). The majority of women received PNC education in a group setting, although district and sub-district level hospitals conducted some one-on-one counseling. Family members or other support persons were infrequently involved in the pre-discharge education process (Table 3). Across hospital types, more mothers stated that they were informed about the PNC visit schedule than could recall it; however, mothers in the sub-district hospitals were significantly more likely to know the correct number of recommended PNC visits and the schedules than were the patients in the regional or district hospitals (*p* < 0.01; Table 3). Only 39 (23%) of the interviewed women stated that they were given a phone number or contact to call if they had questions or concerns following discharge and this was similar across hospitals. Two women, both in sub-district level hospitals, stated that they did not receive PNC instructions. Both were aware of the need for PNC but only one stated she was given a contact number to call for questions.

**Table 2:** Patient demographic data and education

	Regional Hospital	District Hospitals	Sub-district Hospitals
Age (years)	30 ± 5.8	30 ± 6.1	28 ± 4.9
Primiparous	8 (38%)	26 (38%)	24 (29%)
Education			
Uneducated	1 (5%)	3 (4%)	5 (6%)
Primary	2 (10%)	8 (12%)	9 (11%)
Secondary	13 (62%)	39 (57%)	62 (75%)
Tertiary	5 (25%)	12 (18%)	5 (6%)
Not answered	0	6 (8%)	2 (2%)
Total	21	68	83

Data are represented by mean ± SD or number (%). There are no significant differences between groups

**Table 3:** Patient awareness of PNC visits and format of pre-discharge education

	Regional Hospital	District Hospitals	Sub-district Hospitals
Informed of need for PNC during ANC visits	11 (52%)	36 (53%)	51 (61%)
Format of pre-discharge education			
One to one	–	17 (25%)	29 (35%)
Group	21 (100%)	50 (74%)	47 (57%)
Both one to one and group	–	1 (1%)	4 (5%)
Not answered	–	–	2 (2%)
Support person present for pre-discharge education	0	7 (10%)	22 (27%)
Instructed on PNC visit schedule	10 (48%)	45 (66%)	61 (73%)
Knew the correct number of PNC visits	2 (10%)	10 (15%)	35 (42%)*
Could recall the correct PNC visit schedule	0	4 (6%)	29 (35%)*
Informed where to go for PNC visits	15 (71%)	59 (87%)	72 (87%)
Given resource number to call if questions	5 (24%)	10 (15%)	24 (29%)

Data are represented as percent of patients within each hospital category

*Patients in the sub-district hospitals were significantly more likely to know the correct number and timing of PNC visits (*p* < 0.01; chi-square). Postnatal care (PNC)

**Fig. 1:**
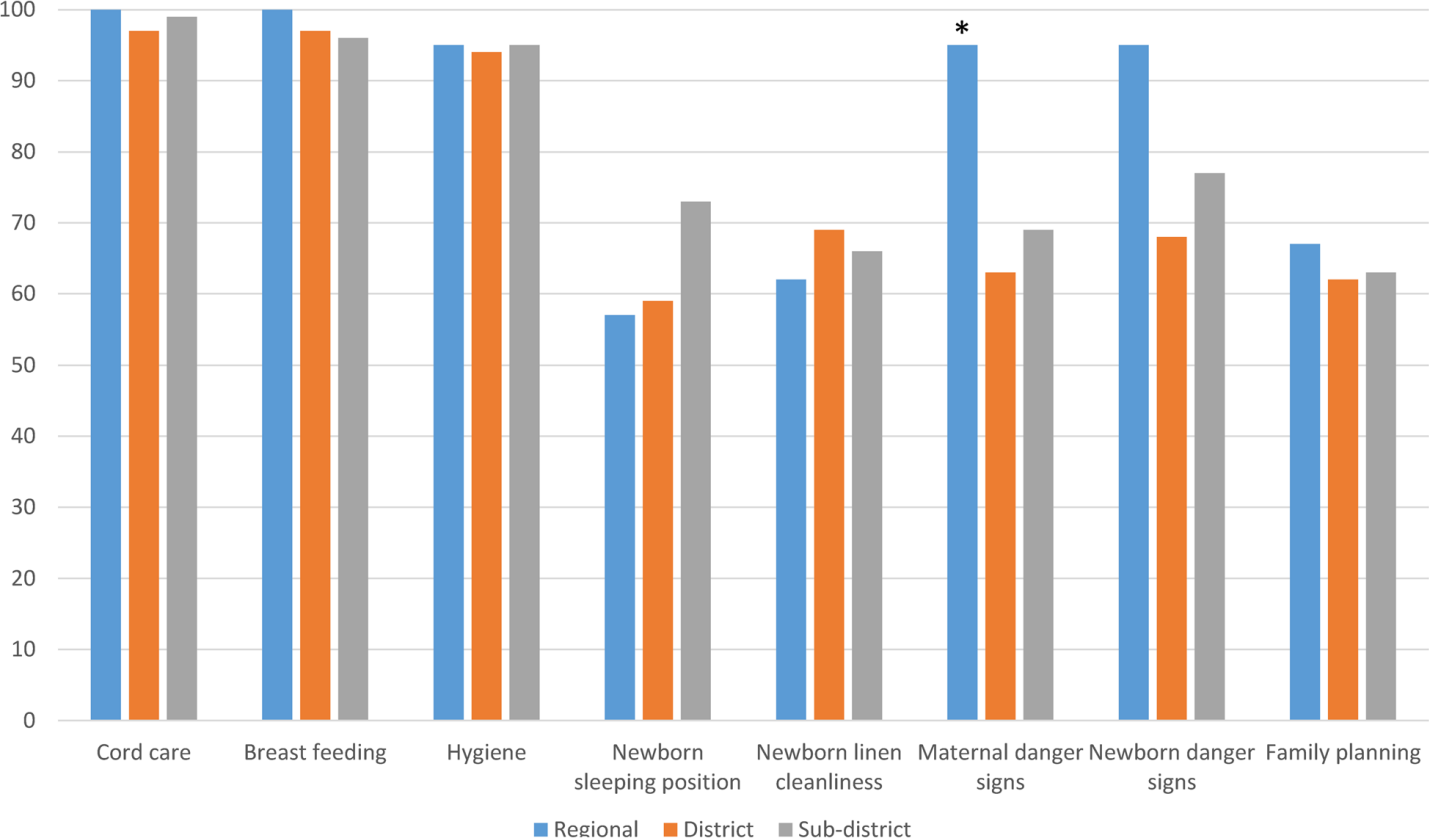
Percent of patients informed on key postnatal topics prior to hospital discharge. Patients in the regional hospital were more likely to have received information on maternal danger signs than were patients in the district and sub-district hospitals (*p < 0.05; chi-square)

Mothers were asked if specific content areas were addressed in their pre-discharge instructions (Fig. 1). Responses were uniformly high on the topics of cord care, breastfeeding and personal hygiene. Other subject content areas were consistently lower, apart from maternal danger signs, which were significantly more likely to be discussed with mothers in the regional hospital (*p* = 0.02; Fig. 1). Consistently, women who delivered in the regional hospital were significantly more likely to recall specific maternal danger signs than were the women who delivered in district and sub-district facilities (*p* < 0.01 value; Table 4). Danger signs were described as conditions in which the mother should report back to the hospital. Overall, women identified 13 maternal danger signs, the leading five of which were: vaginal bleeding (*n* = 92; 53%), malaise (*n* = 48; 28%), dizziness (*n* = 38; 22%), pain (*n* = 36; 21%), and headache (*n* = 28; 16%). Thirty-nine (28%) women reported that they were not informed about maternal danger signs. Similarly, mothers described 18 separate newborn danger signs, the leading five of which were: jaundice (*n* = 77; 45%), fever (*n* = 56; 33%), lethargy (*n* = 40; 23%), rash (*n* = 24; 14%), and cord problems (*n* = 23; 13%). Thirty-one mothers (18%) were unable to name any newborn danger signs. No mother recalled more than four maternal and five newborn danger signs.

**Table 4:** Maternal and newborn danger signs recalled by patients by hospital category

	Maternal Danger Signs			Newborn Danger Signs		
Number recalled	Regional Hospital*	District Hospitals	Sub-district Hospitals	Regional Hospital	District Hospitals	Sub-district Hospitals
0	5	28	24	0	22	19
1	19	16	10	24	34	23
2	14	38	46	38	32	40
3	48	15	18	38	9	13
4	14	3	2	0	3	4
5	0	0	0	0	0	1

Data are presented by the percent of patients who were unable or able to recall one or more maternal or newborn danger signs prior to hospital discharge. *Mothers in regional hospitals were significantly more likely to recall maternal danger signs (*p* < 0.01; chi-square)

**Fig. 2:**
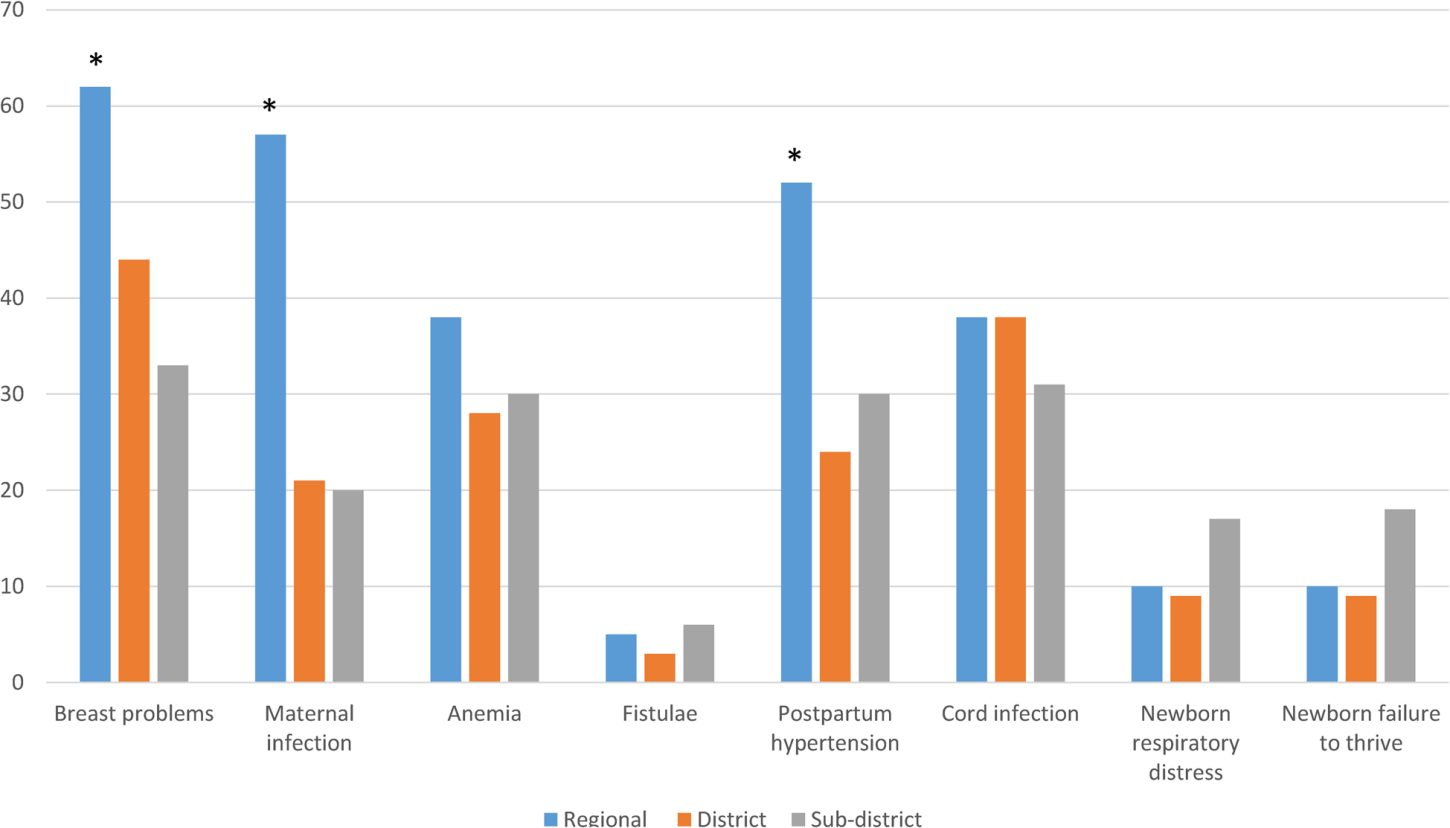
Percent of patients who received information on specific postpartum problems. Breast problems (engorgement or cracked nipples). Maternal infection (fever, lower abdominal pain, foul smelling discharge or feeling sick). Anemia (extreme tiredness and weakness associated with pale lips and palms). Fistulae (urinary or fecal incontinence). Postpartum hypertension (severe headache, blurred vision, convulsions). Cord infection (wet and foul-smelling umbilical cord). Newborn respiratory distress (baby is breathing fast and having an indrawing chest). Newborn failure to thrive (baby is lethargic, poor feeding, has fever or low temperature). Patients in the regional hospital were more likely to have received information on breast problems, maternal infection and postpartum hypertension than were patients in the district and sub-district hospitals (*p < 0.05; chi-square)

Mothers were additionally asked if they were instructed on how to identify important postpartum conditions. Women in regional hospitals were significantly more likely to have received information about recognizing breast problems, signs of maternal infection and postpartum hypertension (*p* < 0.05; Fig. 2); however, the overall rates of information sharing on common complications was low. When mothers were asked the minimum recommended time that newborns be exclusively breastfed, 95% reported 6 months with response rates high among facilities. Eighty-eight percent of mothers felt that their questions and concerns were addressed and this was consistent across facilities. Similarly, mothers graded the adequacy of their postnatal information as 4.2 on a five-point Likert scale (1 = very inadequate, 2 = inadequate, 3 = average, 4 = adequate, 5 = very adequate) with little variation across facility type. When asked what could be improved, 135 (78%) of women had nothing to add; however, eleven specific suggestions were made. In order of frequency, women recommended: more emphasis on danger signs (7), having a ANC/PNC school (6), improving staff relations (6), one-on-one education (4), sex education/family planning (4), breastfeeding education (3), having a take home checklist (2), having a PNC schedule (2), clearer language (1), advice on medical bills/finance (1), and providing all information at one time (1). Responses among women were compared in facilities that reported conducting discharge at six versus 24 h following routine vaginal delivery. There were no differences in responses with regards to the counseling format utilized, instruction received on maternal and newborn danger signs or family planning. Women discharged from facilities practicing early discharge more frequently reported having a family member present during PNC counseling (*p* = .04) whereas women discharged from facilities at 24 h were more likely to recall maternal danger signs (*p* = 0.05).

### Provider interviews

A healthcare provider from 12 of the 13 facilities was also surveyed to gauge their perceptions of postnatal counseling; one district hospital did not participate. Of the 12 providers, four were principle midwifery officers, three were principle nursing officers, three were senior midwifery officers and two were senior staff midwives. Healthcare providers were asked if they had written PNC educational guidelines; five (42%) reported that they did not have written guidelines including one district and four sub-district facilities. In facilities with guidelines, three respondents felt there was room for improvement. When asked what type of counseling format was provided, 83% responded that both a combined group and one-on-one format was provided and 17% reported group sessions only. Sixty-seven percent of interviewed staff recalled the recommended schedule for PNC visits; the regional hospital, one district and 2 sub-district facility respondents could not. Most staff indicated PNC was recommended at one- and six-weeks following delivery; however, there was inconsistency recalling the number and timing of visits within the first 72 h.

All providers indicated that they allowed questions from mothers during counselling sessions and 83% encouraged mothers to call following discharge for any challenges or concerns. Eighty-three percent of providers reported positive views toward the level of PNC education they provided and two facilities (17%), including one district and one sub-district facility, perceived a need for improvement. Only one sub-district provider thought that the information given to mothers was inadequate. The providers were asked what barriers were faced in educating mothers in an open-ended format as well as strategies they would recommend for improvement. The responses were organized into categories based on three themes: language differences (58%), personal issues (33%) such as “patients will do what they want regardless of counseling”, and a lack of written guidelines to follow (17%). When asked to make recommendations to improve PNC counselling, five themes emerged from the providers’ responses: adding take-home material for mothers (33%), establishing standard guidelines (33%), involving relatives in postnatal care visits (25%), giving demonstrations during counselling (17%), and increasing follow-up visits (17%).

## Discussion

This study provides important insights into maternal and provider perspectives on PNC counselling prior to discharge in 13 healthcare facilities in Accra, Ghana. There have been relatively few studies related to the provision of postpartum care in health facilities in LMIC. Recently, Benova and colleagues sampled Demographic and Health Survey data from 33 sub-Saharan African countries [2]. Of the 137,218 women represented in the study, only 66.6% (95% CI: 66.2–67.1) were estimated to have received a pre-discharge check by a health professional [2]. There was considerable variation between countries, with a range from 26.6% in Eswatini (Swaziland) to 94.4% in Burkina Faso. In only four countries, including Burkina Faso, Ivory Coast, Ghana, and Sierra Leone, did more than 90% of women report receiving this check, which is consistent with the present findings. A similar study by Wang also found suboptimal and widely ranging levels of postpartum care among women having facility births ranging from 41% in Uganda to 92% in Ghana, but the study did not assess whether a postnatal check occurred while women were still hospitalized [20]. Neither study addressed the content, quality or format of the postnatal check, which are critical in promoting a greater use of PNC services across the full 6-week postpartum period.

The current study explored the staffing, structure and timing around PNC and pre-discharge patient education. It was encouraging that all but three sub-district level facilities reported having dedicated postnatal nursing staff. Subspecialized nursing care is routine in high income settings, where knowledge and skill set requirements differ tremendously for conditions encountered during the intrapartum, intraoperative or postpartum periods. The presumed benefit of having dedicated nurses is potentially threatened in Ghana; however, by the practice of staff rotation. This practice occurred annually or biennially in all facilities except the regional and three sub-district hospitals. The risk in staff rotation is that newly rotated staff have less experience in the new content area making problem identification, conduct of care, policy and procedure adherence more difficult. The lack of established guidelines in 42% of the surveyed facilities further compounds the problem. The information provided to mothers was not uniform, but this could have been facility dependent. For example, lower level hospitals are more likely to treat uncomplicated pregnancies and explanation of some levels of complications (wound infection) would not be warranted. What is clear is that poor, incomplete, and non-standardized patient information can reduce the quality care [16].

The structure of pre-discharge counseling was most commonly a group format. This format has been shown to be effective in Rwanda [21]. Sub-district hospitals were more likely to offer individual counseling and to involve support persons. Individualized counseling may have been more likely in sub-district facilities due to lower patient volume and complexity, which could have allowed for more staff and patient interaction. Indeed, patients in these facilities were more likely to know the number and schedule of PNC visits. It was concerning that 33% of providers could not correctly recall the PNC schedule; three of the four incorrect responses were from hospitals that did not rotate staff. It is imperative that providers have a clear understanding of the recommended postnatal schedule and share this schedule with mothers. Interestingly, providers reported that the care they gave was more individualized than what the mothers recalled. Another similar inconsistency, was that only 23% of mothers reported being given a resource number to call if they had a problem or concern following discharge, yet 83% of providers responded that this information had been provided. These discrepancies could be attributed to desirability bias in responses given by healthcare providers, inability for mothers to comprehend or recall information due to a state of exhaustion following birth, or both.

The timing of the PNC was also explored prior to discharge. The WHO recommends that women with uncomplicated vaginal births remain in health facilities for at least 24 h to provide health workers the opportunity to detect problems in women and newborns before discharge [12, 13]. The present study found that six (46%) hospitals discharged patients within 6 hours of vaginal delivery. This was true for both district and sub-district level hospitals, negating the argument that this only occurs in high-volume facilities needing rapid turnover. Discharge at 6 hours is problematic as mothers may be exhausted after giving birth, making it difficult to absorb or recall detailed instructions, especially without the presence of family members. Within this timeframe, problems with breastfeeding or infection may also be too early to surface. A recent study showed that the postpartum length of stay in facilities might be too short in many countries to identify and treat complications that contribute to maternal and neonatal mortality in the first 72 h after birth [22, 23]. A 24-h stay could theoretically provide more time for counseling on danger signs, common postpartum problems and family planning, but we did not show any differences in maternal responses given related to their presumed timing of discharge apart from better recall of maternal danger signs in women delivering in hospitals routinely discharging at 24 h.

Although patients indicated satisfaction with the adequacy of counseling and the majority reported that their questions and doubts were addressed, there were deficiencies in key content areas presented. High patient satisfaction levels are common in low resource settings even when care is inadequate [16]. Nearly all mothers reported having received instruction on cord care, breastfeeding and hand and personal hygiene prior to discharge, but the quality of such counseling wasn’t evaluated. Mothers revealed that other important topics, such as the correct sleeping position for a newborn and family planning, were discussed much less frequently by healthcare providers and this was found across institutions. More mothers in the regional hospital stated that they were instructed on, and they could recall, specific maternal danger signs. Similarly, mothers in regional hospitals more commonly reported education on how to recognize breast problems, signs of maternal infection and postpartum hypertension. This is appropriate given that mothers in regional hospitals are more likely to have high risk or complicated pregnancies and making them more prone to developing specific complications such as incisional infection, postpartum hypertension, and premature birth [24]. Many of these common postpartum problems, however, could likely occur in women irrespective of the level of delivery facility and should be included in discharge counseling.

We were encouraged that all but two women (99%) in our patient sample reported receiving PNC education prior to discharge. A recent study by Benova and colleagues conducted among 137,218 post-partum women across 33 sub-Saharan African countries reported that the estimated percentage of women receiving pre-discharge PNC was 67%, with significant variation ranging from 27% in Eswatini (Swaziland) to 94% in Burkina Faso [23]. Consistent with the current findings, Benova found that in Ghana the pre-discharge PNC was over 90% [23]. Regarding maternal knowledge of newborn danger signs, our findings were consistent with a study by Degefa and colleagues from Ethiopia [6]. Degefa reported that 41% of mothers were able to list two or more newborn dangers signs, which they deemed as “good knowledge”, compared to 55% in the present study [6]. Diarrhea (41%) was the most frequently mentioned newborn danger sign in their study versus jaundice (45%) in the present study but both studies found fever to be the second most commonly mentioned danger sign in 33% of women [6]. A qualitative study from Tanzania similarly found that the lack of standardized PNC guidelines was problematic for midwives [10].

There are several limitations to the current study. First, women represented in the study delivered in an urban setting which may not be representative of more rural parts of the country. It has been shown that women who are younger, poorer, rural and of higher-parity are less likely to receive pre-discharge PNC [11, 23]. Secondly, women were not asked about their mode of delivery or timing of discharge which would have allowed for more detailed analysis. Third, it is unclear whether women already knew about danger signs, newborn and self-care prior to delivery or whether these details were learned at pre-discharge PNC. Fourth, we were unable to determine the quality of PNC presented to mothers. Despite these limitations, disparities are evident in Ghanaian PNC and there are ample opportunities to bridge these gaps. A common suggestion for improvement offered by both mothers and providers in this study is a desire for written guidelines and take-home discharge materials for mothers. A postnatal educational brochure was developed based on these suggestions, in conjunction with the Ghana Health Service, but the time remaining within the project did not allow the opportunity to study its usefulness. Given that the majority of mothers in this urban setting reported having secondary or higher forms of education, they would be expected to be able to read, but this might not be the case in other patient populations. It would be helpful to repeat the patient interviews to better understand the knowledge retained by mothers after receiving the educational materials. We would encourage the Ghana Health Service to reproduce and distribute the post-natal discharge instructions generated through this program.

## Conclusion

The present study addresses the content, quality and format of postnatal pre-discharge care and counseling across a cluster of institutions in the Greater Accra region of Ghana, Pre-discharge counseling is critical in the promotion of a greater use of PNC services across the full 6-week postpartum period. The gaps identified in the study include over reliance on a group counselling format, staff unfamiliarity with standard postnatal visit schedules, and discrepancies between the information purported to have been given during the postnatal counselling sessions and what is actually remembered by the patients. Measures that can help address these issues include, but are not limited to, the introduction of a standard postnatal counselling checklist, the development of postnatal educational brochures to be taken home by the patients, the provision of staff with adequate knowledge and skills in effective counselling, It is important to recognize and treat problems early to prevent serious complications and death. By strengthening postnatal care and education throughout Ghana, it is hoped that a wider demand will be generated for high quality postnatal care that can provide mothers and babies a better chance at survival in the critical postnatal period.

## Supplementary Information

**Additional file 1.**

**Additional file 2.**

## Data Availability

The datasets generated and/or analysed during the current study are available from the corresponding author on reasonable request.

## Abbreviations

ANC: Antenatal care
GARH: Great Accra Regional Hospital
LMIC: Low- and middle-income countries
PNV: Postnatal care
WHO: World Health Organization

## References

[1] Campbell OM, Graham WJ (2006). Lancet maternal survival series steering g. strategies for reducing maternal mortality: getting on with what works. Lancet..

[2] Benova L, Tuncalp O, Moran AC, Campbell OMR (2018). Not just a number: examining coverage and content of antenatal care in low-income and middle-income countries. BMJ Glob Health.

[3] Tikmani SS, Ali SA, Saleem S, Bann CM, Mwenechanya M, Carlo WA (2019). Trends of antenatal care during pregnancy in low- and middle-income countries: findings from the global network maternal and newborn health registry. Semin Perinatol.

[4] Arunda M, Emmelin A, Asamoah BO (2017). Effectiveness of antenatal care services in reducing neonatal mortality in Kenya: analysis of national survey data. Glob Health Action.

[5] Browne JL, Kayode GA, Arhinful D, Fidder SA, Grobbee DE, Klipstein-Grobusch K (2016). Health insurance determines antenatal, delivery and postnatal care utilisation: evidence from the Ghana demographic and health surveillance data. BMJ Open.

[6] Degefa N, Diriba K, Girma T, Kebede A, Senbeto A, Eshetu E (2019). Knowledge about neonatal danger signs and associated factors among mothers attending immunization Clinic at Arba Minch General Hospital, southern Ethiopia: a cross-sectional study. Biomed Res Int.

[7] Ntambue AM, Malonga FK, Dramaix-Wilmet M, Ngatu RN, Donnen P (2012). Determinants of maternal health services utilization in urban settings of the Democratic Republic of Congo—a case study of Lubumbashi City. BMC Pregnancy Childbirth.

[8] Lawn JE, Lee AC, Kinney M, Sibley L, Carlo WA, Paul VK (2009). Two million intrapartum-related stillbirths and neonatal deaths: where, why, and what can be done?. Int J Gynaecol Obstet.

[9] UNICEF W (2017). World bank group, United Nations. Levels and trends in child mortality: report 2017.

[10] Dol J, Kohi T, Campbell-Yeo M, Tomblin Murphy G, Aston M, Mselle L (2019). Exploring maternal postnatal newborn care postnatal discharge education in Dar Es Salaam, Tanzania: barriers, facilitators and opportunities. Midwifery..

[11] Langlois EV, Miszkurka M, Zunzunegui MV, Ghaffar A, Ziegler D, Karp I (2015). Inequities in postnatal care in low- and middle-income countries: a systematic review and meta-analysis. Bull World Health Organ.

[12] Organization WH (2013). WHO recommendations on postnatal Care of the Mother and Newborn.

[13] Organization WH (2018). WHO recommendations: Intrapartum Care for a Positive Childbirth Experience.

[14] Ghana Statistical Service GHS, ICF International (2015). Ghana demographic and health survey, 2014. Rockville.

[15] Hill Z, Okyere E, Wickenden M, Tawiah-Agyemang C (2015). What can we learn about postnatal care in Ghana if we ask the right questions? A qualitative study. Glob Health Action.

[16] Kruk ME, Gage AD, Arsenault C, Jordan K, Leslie HH, Roder-DeWan S (2018). High-quality health systems in the sustainable development goals era: time for a revolution. Lancet Glob Health.

[17] Gabrysch S, Nesbitt RC, Schoeps A, Hurt L, Soremekun S, Edmond K (2019). Does facility birth reduce maternal and perinatal mortality in Brong Ahafo, Ghana? A secondary analysis using data on 119 244 pregnancies from two cluster-randomised controlled trials. Lancet Glob Health.

[18] Ghana Population 2020 (Live) 2020 [Available from: https://worldpopulationreview.com/countries/ghana-population/.

[19] Bailey PE, Awoonor-Williams JK, Lebrun V, Keyes E, Chen M, Aboagye P (2019). Referral patterns through the lens of health facility readiness to manage obstetric complications: national facility-based results from Ghana. Reprod Health.

[20] Wang W, Alva S, Wang S, Fort A (2011). DHS comparative reports no. 26. Levels and trends in the use of maternal health Services in Developing Countries. Calverton, Maryland.

[21] Lundeen T, Musange S, Azman H, Nzeyimana D, Murindahabi N, Butrick E (2019). Nurses’ and midwives’ experiences of providing group antenatal and postnatal care at 18 health centers in Rwanda: a mixed methods study. PLoS One.

[22] Campbell OM, Cegolon L, Macleod D, Benova L (2016). Length of stay after childbirth in 92 countries and associated factors in 30 low- and middle-income countries: compilation of reported data and a cross-sectional analysis from nationally representative surveys. PLoS Med.

[23] Benova L, Owolabi O, Radovich E, Wong KLM, Macleod D, Langlois EV (2019). Provision of postpartum care to women giving birth in health facilities in sub-Saharan Africa: a cross-sectional study using demographic and health survey data from 33 countries. PLoS Med.

[24] Ramaswamy R, Kallam B, Kopic D, Pujic B, Owen MD (2016). Global health partnerships: building multi-national collaborations to achieve lasting improvements in maternal and neonatal health. Glob Health.

