# Supplementary material for: Postnatal care education in health facilities in Accra, Ghana: perspectives of mothers and providers
Source: BMC Pregnancy Childbirth. 2020 Nov 4;20:664. doi: 10.1186/s12884-020-03365-1 (PMC7640641; doi:10.1186/s12884-020-03365-1)
Supplement: Supplementary file 1 — Additional file 1. [file 12884_2020_3365_MOESM1_ESM.docx]

**Exit Interview of Post-Natal Mothers about the Content of Post-natal Care**

1. Age : _______________________________________________________
2. Parity : _______________________________________________________
3. Educational status : _______________________________________________________
4. Occupation : _______________________________________________________
5. Where do you stay? : _______________________________________________________
6. Name of institution of delivery: _____________________________________________________
7. Which facility where you referred from: ______________________________________________


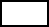
8. Were you informed about the importance of Post-natal care during the antenatal period? Yes No

9. Did you receive postal natal counselling before being discharged? Yes
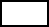
 No
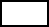


10. Nature of counselling:

a. one-to-one only
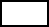


b. group counselling only
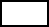


c. both one to one and group
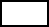


11. Were your family members involved in the counselling session? Yes
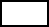
 No
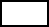


12. If Yes, who were the people present? _________________________________________________

13. Where were you asked to have your postnatal visit? _____________________________________

14. Were you given information on the following?

|  | Yes | No |
| --- | --- | --- |
| Cord care |  |  |
| Breast Feeding |  |  |
| Personal and Hand Hygiene |  |  |
| How and where the baby should sleep |  |  |
| Keeping cot sheets clean and ironed |  |  |
| Wound care |  |  |
| Dangers signs and symptoms of mother for which you should medical attention immediately |  |  |
| Dangers signs and symptoms of baby for which you should medical attention immediately |  |  |
| Family Planning |  |  |
| Post-natal visit schedules |  |  |

15. What should be the minimum number of postnatal visits even if all things are fine? ______________

16. State the visit schedules ______________________________________________________________

17. Is there any reason for which you could be unable to report for post-natal visit at all?

Yes
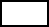
 No
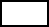
 If yes, mention a few ____________________________

_____________________________________________________________________________________

18. Mention as many Dangers signs and symptoms of **mother** that you were told for which you should immediately get back to the hospital ______________________________________________________

____________________________________________________________________________________

19. Mention as many Dangers signs and symptoms of **baby** that you were told for which you should report immediately get back to the hospital _________________________________________________

_____________________________________________________________________________________

20. Were the following mentioned?

| **CONTENT** | Yes | No |
| --- | --- | --- |
| Exclusive bread feeding for six months? |  |  |
| Breast problems (engorgement, cracked nipples)? |  |  |
| Fever, lower abdominal pain, foul smelling discharge or feeling sick as a sign of infection? |  |  |
| Extreme tiredness and weakness associated with pale lips and palms as a sign of anemia? |  |  |
| Heavy vaginal bleeding? |  |  |
| Urine or fecal incontinence (obstetric fistula)? |  |  |
| Severe headaches, blurred vision, convulsions may be associated with post-partum hypertension |  |  |
| Were you taught how to care for the cord and avoid putting various non-prescription materials on it? |  |  |
| Were you told the significance if the cord is wet and offensive? |  |  |
| Were you told the significance if the child is breathing fast and has chest in-drawings? |  |  |
| Were you told the significance of lethargy, poor feeding, failure to thrive, fever, and low temperature? |  |  |

21. When roughly after delivery of this baby did you put the baby to the beast (what is the standard recommendation)?_____________________________________________________________________

22. How long should a baby be breast fed exclusively? ________________________________________

23. Do you have a resource number to call if you have a question or a concern to be addressed?

Yes
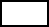
 No
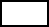


24. Do you think all your questions and doubts were addressed during the counseling session?

Yes
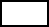
 No
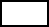


25. How will you grade the adequacy of the educational information given you after delivery

1. Very inadequate
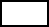


2. Inadequate
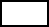


3. Average
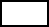


4. Adequate
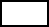


5. Very adequate
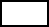


26. What would you suggest to be improved with regards to the post-natal education given? _______________________________________________________________________________________________________________________________________________________________________________________________________________________________________________________________

***Thank you for your participation***
