# Supplementary material for: Postnatal care education in health facilities in Accra, Ghana: perspectives of mothers and providers
Source: BMC Pregnancy Childbirth. 2020 Nov 4;20:664. doi: 10.1186/s12884-020-03365-1 (PMC7640641; doi:10.1186/s12884-020-03365-1)
Supplement: Supplementary file 2 — Additional file 2. [file 12884_2020_3365_MOESM2_ESM.docx]

**KEY INFORMANT INTERVIEW GUIDE FOR MATERNITY IN-CHARGE**

1. Health Facility Name: ________________________________________________________
2. Staff Grade: ________________________________________________________________
3. Do you have written PNC education guidelines? Yes No

(checklist, job aid or chart used to give mothers health talk and demonstration) Ask to see.

1. What do you use? ___________________________________________________________
2. Nature of counseling:
3. One-to-one only
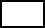

4. Group counseling only
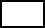

5. Both one-to-one and group
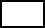

6. Do you allow questions from the mothers during postnatal sessions? Yes
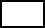
 No
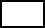

7. What is the schedule for postnatal visit? __________________________________________
8. Are mothers encouraged to call when they have a challenge or concern? Yes
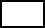
 No
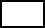

9. Are newborns given antibiotic eye drops? Yes
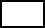
 No
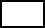

10. In your opinion and informed by your experience, what is the level of adherence to PNC guidelines by your staff? ______________________________________________________
11. Generally, what is your view with regards to the level of PNC education (health talk, demonstration, advice etc.) given to the mothers in this unit before discharge from maternity? _____________________________________________________________________________
12. Do you feel that the information, advice or demonstration you give to the mothers is adequate? Please expain__________________________________________________________________
13. What barriers/challenges do you encounter in educating the mothers? Please elaborate_____________________________________________________________________
14. What strategics and interventions would you recommend to improve PNC education in the maternity unit in the future? Please provide justification_____________________________
15. Is there anything else you would like to add? ______________________________________

___________________________________________________________________________

***Thank you for your participation***
